# Coding regions affect mRNA stability in human cells

**DOI:** 10.1261/rna.073239.119

**Published:** 2019-12

**Authors:** Ashrut Narula, James Ellis, J. Matthew Taliaferro, Olivia S. Rissland

**Affiliations:** 1 Program in Molecular Medicine, The Hospital for Sick Children, Toronto, Ontario M5G 0A4, Canada; 2 Program in Developmental and Stem Cell Biology, The Hospital for Sick Children, Toronto, Ontario M5G 0A4, Canada; 3 Department of Molecular Genetics, University of Toronto, Toronto, Ontario M5S 1A8, Canada; 4 RNA Bioscience Initiative and Department of Biochemistry & Molecular Genetics, University of Colorado School of Medicine, Aurora, Colorado 80045, USA

**Keywords:** codons, post-transcriptional regulation, translation elongation, mRNA stability

## Abstract

A new paradigm has emerged that coding regions can regulate mRNA stability in model organisms. Here, due to differences in cognate tRNA abundance, synonymous codons are translated at different speeds, and slow codons then stimulate mRNA decay. To ask if this phenomenon also occurs in humans, we isolated RNA stability effects due to coding regions using the human ORFeome collection. We find that many open reading frame (ORF) characteristics, such as length and secondary structure, fail to provide explanations for how coding regions alter mRNA stability, and, instead, that the ORF relies on translation to impact mRNA stability. Consistent with what has been seen in other organisms, codon use is related to the effects of ORFs on transcript stability. Importantly, we found instability-associated codons have longer A-site dwell times, suggesting for the first time in humans a connection between elongation speed and mRNA decay. Thus, we propose that codon usage alters decoding speeds and so affects human mRNA stability.

## INTRODUCTION

Transcript destruction is key for controlling gene expression. Messenger RNA (mRNA) decay not only helps set the overall expression level of genes, but it also determines the dynamics of their expression. Unsurprisingly, proper control of mRNA decay is critical in diverse biological processes ranging from the maternal-to-zygotic transition to the inflammatory response (Giraldez et al. 2006; Benoit et al. 2009; Brooks and Blackshear 2013). A major line of research has thus been to understand both the elements within an RNA that control its stability as well as the mechanisms underlying this regulation.

For decades, researchers have focused on the 3′ untranslated regions (3′UTRs) for several important reasons. First, especially in humans, 3′UTRs contain extensive sequence space that has large regulatory potential while also being devoid of other constraints (such as encoding a protein). Second, regulatory factors, such as RNA binding proteins (RBP), can stably bind the 3′UTR, which is not exposed to the translating ribosome, unlike the 5′UTR or open reading frame (ORF) (Grimson et al. 2007). Thus, elements within the 3′UTR, such as those bound by microRNAs (miRNAs), provide our best understanding of the mechanisms and regulation of mRNA decay (Rissland 2016). Briefly, sites in the 3′UTR are recognized by regulatory factors, such as RBP or miRNAs. These regulatory factors both remove stabilizing protein components and also recruit deadenylase complexes, which then shorten the poly(A) tail found on nearly all eukaryotic transcripts (Eulalio et al. 2009; Fabian et al. 2009, 2011; Zekri et al. 2013; Kuzuoğlu-Öztürk et al. 2016; Rissland et al. 2017). Following deadenylation, the decapping enzyme then removes the 5′ cap, thereby exposing the body of the transcript to the major cytoplasmic 5′ → 3′ exonuclease, Xrn1 (Yamashita et al. 2005; Chen et al. 2009).

In contrast, outside of surveillance pathways, our understanding of how the coding region influences mRNA stability lags behind that of 3′UTR-based regulation. Nonetheless, over the past few years, research in a number of model organisms has demonstrated that coding regions play a major role in mRNA stability. Best defined by work in *Saccharomyces cerevisiae*, the primary mechanism by which ORFs regulate mRNA stability is through codon usage (Presnyak et al. 2015). Although synonymous codons encode the same amino acid, they are not equivalent to the elongating ribosome. Due to differences in tRNA abundance, demand, and wobble interactions, some synonymous codons are decoded faster than others (Pechmann and Frydman 2013; Gardin et al. 2014; Yu et al. 2015; Hanson et al. 2018; Wu et al. 2019a). These differences in elongation speed are sensed by the decay machinery, resulting in recruitment of the CCR4-NOT deadenylase complex, the decapping activator Dhh1p, and eventual decapping (Radhakrishnan et al. 2016; Webster et al. 2018). In other words, mRNAs with low optimality codons (i.e., those decoded slowly) are more unstable than those with high optimality codons. Although initially made in budding yeast, these observations have now been extended to *Escherichia coli*, *Schizosaccharomyces pombe*, trypanosomes, and metazoans (Bazzini et al. 2016; Boël et al. 2016; Harigaya and Parker 2016; Mishima and Tomari 2016; Mattijssen et al. 2017; Burow et al. 2018; de Freitas Nascimento et al. 2018; Jeacock et al. 2018; Wu et al. 2019b), likely pointing to a conserved evolutionary mechanism.

An important unanswered question has been the extent to which these observations hold in humans. This question has been raised in part because human 3′UTRs are often longer than 1000 nt (Lianoglou et al. 2013), which has led to the model that 3′UTRs are the dominant regulatory force for post-transcriptional regulation in humans. Importantly, all elements of a gene, including the coding region and UTRs, coevolve, and so exploring the contribution of codons to endogenous mRNA stability is confounded by UTR effects. Another barrier has been a lack in the consensus of which codons mediate fast elongation speeds in human cells, and so calling codons as “optimal” or “nonoptimal” has been challenging. The lack of consensus has come about for at least three reasons. First, unlike in yeast, only slight relationships have been observed between translation elongation speeds and standard metrics of codon optimality, such as tRNA abundance and codon adaptivity index (Ingolia et al. 2011; Darnell et al. 2018). Second, tRNA abundance is notoriously hard to measure due to extensive RNA modifications and secondary structure. Highly processive reverse transcriptases, like TGIRT, and other high-throughput sequencing methods have enabled better quantitation, but measurements from different labs still fail to correlate well, even when performed on the same cell type (Zheng et al. 2015; Gogakos et al. 2017; Mattijssen et al. 2017). Third, tRNA abundance also differs between different cell types and states (Dittmar et al. 2006; Goodarzi et al. 2016; Shigematsu et al. 2017; Torrent et al. 2018), leaving open the possibility that some variation in tRNA measurements and elongation speeds reflect biological differences.

To understand how the coding region could impact mRNA stability in humans, we turned to the human ORFeome collection, which contains full-length human ORFs surrounded by invariant UTRs. By using this collection as a parallel reporter system, we isolated the effects of the coding region on mRNA stability. We found that different ORFs mediate a wide range of mRNA stabilities and that the majority of their impact depends on translation. Similar to results from model organisms and a parallel study in humans (Bazzini et al. 2016; Harigaya and Parker 2016; Mishima and Tomari 2016; Burow et al. 2018; de Freitas Nascimento et al. 2018; Jeacock et al. 2018; Wu et al. 2019b), our results point to codon usage as a key feature of the ORF that alters mRNA stability. Moreover, our analyses revealed that codons associated with instability have longer A-site dwell times, thus providing a link between elongation speeds and mRNA decay in humans.

## RESULTS

### Changing the coding region can change mRNA stability in human cells

Given recent reports on the influence of codon use on mRNA stability, we wanted to explore the potential of the ORF to impact mRNA stability in human cells. However, codon use and other ORF features coevolve with mRNA characteristics (such as length, translational efficiency, 3′UTR regulation), all of which have the potential to impact mRNA stability (Schnall-Levin et al. 2011; Duan et al. 2013; Geisberg et al. 2014; Presnyak et al. 2015; Neymotin et al. 2016). We reasoned that these confounding factors would significantly impact genome-wide analyses, making it challenging to disentangle correlative and causative features. To explore this question, we thus turned to the human ORFeome collection because it contains thousands of coding regions surrounded by invariant UTRs as lentiviral constructs.

We decided to use the ORFeome collection as a parallel reporter system and split the collection into six pools, which we then used to generate six corresponding sets of pooled stable HEK293T cell lines through lentiviral infection. Western blotting against the V5 tag at the carboxy terminus of each ORFeome construct confirmed that many ORFs were expressed after stable line generation (Supplemental Fig. S1A). We then used approach-to-equilibrium 4-thiouracil (4SU) metabolic labeling followed by biotinylation and streptavidin pull-down to measure mRNA stability transcriptome-wide (Lugowski et al. 2017).

**FIGURE 1.**
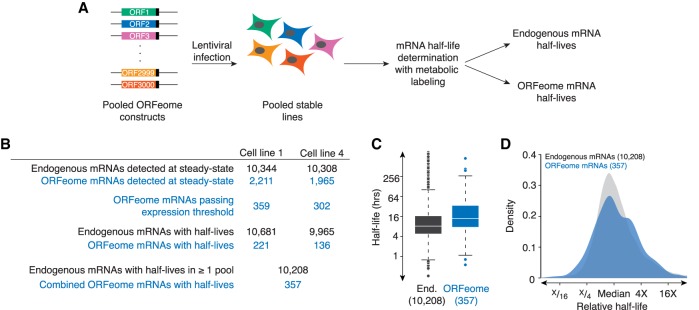
Coding sequences regulate mRNA stability in human cells. (*A*) Schematic of ORFeome pool creation. The ORFeome collection contains ∼15,000 full-length coding regions in a lentiviral plasmid background, where each ORF is flanked by invariant UTRs and also contains a carboxy-terminal V5 tag. Pools of ∼3000 ORFeome clones from the collection were used to make lentivirus, which was then used to infect HEK293T cells and generate pools of stable cell lines. After selection, mRNA half-lives were measured through an approach-to-equilibrium 4SU-labeling experiment, giving stabilities for endogenous and ORFeome-derived transcripts. (*B*) Table summarizing the number of endogenous and ORFeome mRNAs passing each step in the processing pipeline. Endogenous and ORFeome transcripts were first examined in the steady state libraries. ORFeome transcripts were required to be expressed >3 times as much in the appropriate cell line than in the other. Once classified as “endogenous” or “ORFeome,” mRNA half-lives were calculated from the metabolic labeling experiment. Total endogenous mRNA half-lives correspond to the number of mRNAs with at least one measured half-life; if more than one half-life was measured, the arithmetic mean was used. (*C*) Coding regions change mRNA stability. Box-and-whisker plots of the stabilities of endogenous (End., in gray) and ORFeome mRNAs (in blue). Line represents median, box demarcates second and third quartiles, points are outliers. (*D*) ORFeome mRNAs show as much variability in stability as endogenous mRNAs. Plotted are the density distributions of median-centered stabilities of endogenous and ORFeome mRNAs (in gray and blue, respectively).

We did not use ORFeome-targeted RNA sequencing, and so we simultaneously captured stabilities for both ORFeome-derived and endogenous mRNAs. However, because very few reads mapped to regions that could be unambiguously defined as ORFeome-derived (i.e., mapping to the junctions between the UTRs and coding region), these reads could not be used to generate half-lives. To enable half-life calculation of the ORFeome-transcripts, we developed a computational pipeline to classify each mRNA (and corresponding half-life) as “ORFeome” or “endogenous” (Fig. 1B). Briefly, reads were mapped to the RefSeq coding regions. To classify an mRNA as “ORFeome,” we required three times more reads in the corresponding cell line in our steady-state libraries than in the paired cell line. We first analyzed the experiment derived from cell lines expressing pools 1 and 4. Of the 2211 pool 1 ORFs that were detected in cell line 1, only 359 were expressed three-times as much in cell line 1 than in cell line 4 (presumably because some were expressed endogenously, while others were not expressed at high enough levels). Similarly, 302 pool 4 ORFs passed this threshold (Fig. 1B). As expected based on this criterion, pool 1 mRNAs were significantly more highly expressed in pool 1 cells, and pool 4 mRNAs, in the pool 4 cells (Supplemental Fig. S1B,C). Coding regions contained in the ORFeome pool 1 that did not cross this threshold were excluded from downstream analysis. We then calculated half-lives for three sets of mRNAs (pool 1, pool 4, and endogenous). Some ORFeome mRNAs, despite passing our initial cut-off, were still lowly expressed, and thus we could not calculate half-lives for all of the ORFeome mRNAs. In total, we obtained half-lives for 357 ORFeome mRNAs (221 from pool 1 and 136 from pool 4), and 10,208 endogenous mRNAs (Supplemental Tables S1, S2).

Having thus obtained half-lives for 357 ORFeome reporters, we next asked whether changing the coding region was sufficient to change mRNA stability. In general, ORFeome mRNAs were more stable than endogenous mRNAs (median half-life: 8.2 h for endogenous mRNAs versus 13.8 h for ORFeome mRNAs; *P* < 10^−15^, Kolmogorov–Smirnov test; Fig. 1C), likely due to the WPRE stabilizing element included in the invariant 3′UTR of the ORFeome collection (Zufferey et al. 1999). Strikingly, despite containing invariant UTRs, the ORFeome mRNAs showed similar variation in stability as endogenous transcripts: half-lives ranged from 0.25 h to >100 h for endogenous mRNAs, and from 0.56 h to >100 h for ORFeome mRNAs (Fig. 1C,D). This result held even when we subsampled endogenous mRNAs to match sample sizes between the two groups (data not shown).

To see whether this result held more broadly, we turned to our other cell lines, obtaining half-lives for (i) 357 ORFeome mRNAs from pools 2 and 3 and (ii) 384 ORFeome mRNAs from pools 5 and 6 (Supplemental Tables S1, S3, S4). Similar to what we observed with pools 1 and 4, the ORFeome mRNAs had similar variation in stabilities as endogenous ones (Supplemental Fig. S1E). Finally, we analyzed a complementary ORFeome data set from Wu et al. (2019b). This data set allowed us to directly compare the variability in decay rates for the same ORFs expressed as endogenous or ORFeome-derived transcripts (Supplemental Fig. S1F). Importantly, there was a similar range in stabilities for these matched ORFeome and endogenous transcripts. Thus, we conclude that the coding region can regulate mRNA stability in human cells. One surprising implication of this result is that merely changing the coding region can give a similar range of stabilities seen with endogenous mRNAs, which differ not only in their coding regions, but also in their UTRs.

### The effects of the coding region on mRNA stability depend predominantly on ribosome loading

The primary function of the coding region of a transcript is to be read by the ribosome, and it has become increasingly clear that translation impacts mRNA stability not just through surveillance pathways, but also through normal decay. Given the connection between translation, the coding region, and mRNA stability, we next asked whether the effects of the coding region on mRNA stability required ribosome loading. To do so, we repeated our ORFeome and endogenous stability measurements (in cell lines 1 and 4), but this time cells were treated either with DMSO or the translation inhibitor 4EGI-1, which disrupts eIF4F and so blocks initiation (Moerke et al. 2007).

**FIGURE 2.**
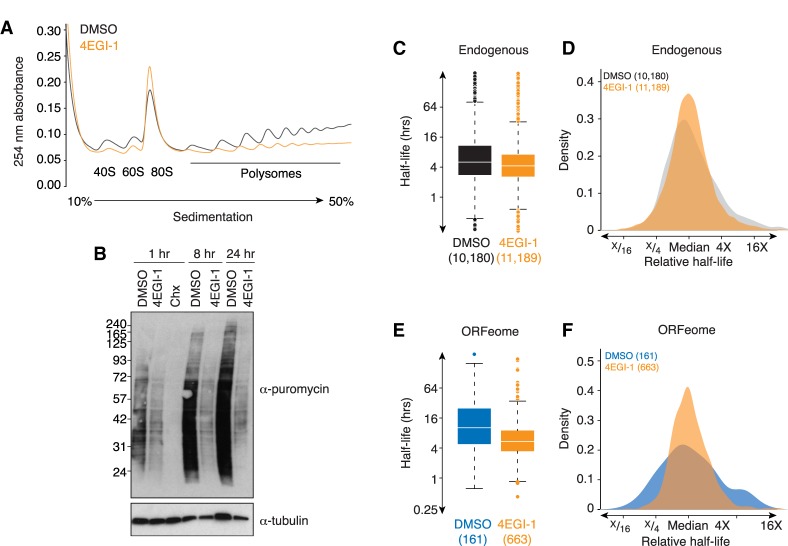
Inhibiting ribosome loading reduces the effects of the coding region on mRNA stability. (*A*) 4EGI-1 treatment inhibits translation. Shown are A254 traces from sucrose density gradients of lysates from HEK293T cells treated with DMSO (gray) or 4EGI-1 (orange). (*B*) 4EGI-1 treatment reduces translation, as measured by puromycin incorporation. Cells were treated with DMSO, 4EGI-1, or cycloheximide (CHX) for the indicated times. To measure ongoing translation, cells were then pulsed with puromycin and harvested. Cell lysates were separated by SDS-PAGE electrophoresis, and western blotting was performed, probing against puromycin and α-tubulin (as a loading control). (*C*) Translation inhibition destabilizes endogenous mRNAs. Plotted are boxplots of half-lives for endogenous HEK293T mRNAs with DMSO or 4EGI-1 treatment (in gray and orange, respectively). The line represents median half-life, the box demarcates second and third quartiles, and points are outliers. (*D*) Translation inhibition has a minor effect on the variation in stability for endogenous mRNAs. Plotted are density distributions of median-centered half-lives for endogenous HEK293T in cells treated with DMSO or 4EGI-1 (in gray and orange, respectively). (*E*) Translation inhibition destabilizes ORFeome-derived mRNAs. As in *C*, except for ORFeome mRNAs. DMSO, in blue; 4EGI-1, in orange. (*F*) Translation inhibition reduces the variation in stability for ORFeome-derived mRNAs. As in *D*, except for ORFeome mRNAs. DMSO, in blue; 4EGI-1, in orange. See also Supplemental Figure S2 and Supplemental Tables S1, S2.

We first confirmed that 4EGI-1 reduced translation by analyzing polysomes by sucrose gradient fractionation and measuring puromycin incorporation followed by western blotting (Fig. 2A,B). As expected, 4EGI-1 treatment robustly reduced translation. (Note that complete inhibition of translation was incompatible with mRNA stability measurements.) 4EGI-1 treatment had broad effects on mRNA stability, both for endogenous and ORFeome mRNAs. Endogenous mRNAs were globally less stable in the presence of the inhibitor (Fig. 2C), consistent with previous studies where translational inhibition destabilized mRNA (Schwartz and Parker 1999). While there was strong correlation for endogenous mRNAs measured in our original experiment and in the presence of DMSO (Spearman r [*r*_*s*_] = 0.67, *P* < 10^−15^, Supplemental Fig. S2), there was only a modest correlation with those measured in the presence of 4EGI-1 (*r*_*s*_ = 0.34, *P* < 10^−15^, Supplemental Fig. S2). Surprisingly, the variation in mRNA decay rates was significantly, albeit modestly, reduced in the presence of 4EGI-1 (σ^2^ 4EGI-1/σ^2^ DMSO = 0.65, *P* < 10^−15^, Fig. 2D).

We saw similar results with the ORFeome mRNAs, although the effects were more pronounced here. Like the endogenous mRNAs, ORFeome mRNAs were also less stable in the presence of 4EGI-1 (*P* < 10^−10^, Kolmogorov–Smirnov test; Fig. 2E). More strikingly, there was substantially less variation in the stabilities of ORFeome mRNAs in the presence of 4EGI-1 (σ^2^ 4EGI-1/σ^2^ DMSO = 0.40, *P* < 10^−1^^5^, Fig. 2F). These results thus suggest that coding region predominantly requires translation to impact stability. It may be that the reduced variability in the stabilities of endogenous mRNAs reflects the contribution of coding regions to endogenous transcript stability. In addition, because ribosome loading was incompletely blocked and inhibiting translation also exposes previously inaccessible regulatory sites in the coding region (see below), the differences observed with 4EGI-1 are likely a lower bound for the influence of translation of the coding region on mRNA decay.

### Length, secondary structure, and RNA binding proteins cannot explain the effects on mRNA stability mediated by the coding region

We next wanted to identify elements within the coding region that influenced RNA stability. Many different ORF features have been associated with mRNA stability: transcript or ORF length; local secondary structure; 3′UTR-like regulation; and codon usage (Schnall-Levin et al. 2011; Duan et al. 2013; Geisberg et al. 2014; Presnyak et al. 2015; Neymotin et al. 2016). For those ORF elements directly regulating mRNA stability, we expected that their signal would be strengthened in the ORFeome data set (compared with the endogenous one) because confounding UTR effects are removed in the ORFeome measurements. In contrast, for elements that coevolve with true causative features, but do not directly affect stability, we expected that their signals would be weaker in the ORFeome data set than in the endogenous one.

We first investigated ORF length in our data from cell lines 1 and 4. Consistent with many previous observations (Duan et al. 2013; Neymotin et al. 2016), for endogenous mRNAs, there was a negative correlation between ORF length and mRNA stability such that transcripts with longer coding regions were more unstable (*r*_*s*_ = −0.14, *P* < 10^−15^, Supplemental Fig. S3A). In contrast, we observed no correlation between ORF length and mRNA stability for ORFeome transcripts (*r*_*s*_ = 0.01, *P* = 0.9, Supplemental Fig. S3). We observed similar results in our other ORFeome pools and the complementary published ORFeome data (Wu et al. 2019b), where the correlation between length and stability was stronger for endogenous transcripts than for ORFeome-derived ones; (endogenous *r*_*s*_: −0.30 to −0.15 versus ORFeome *r*_*s*_: −0.22 to 0.01; Supplemental Fig. S3B). Thus, we conclude that ORF length, at most, only weakly affects mRNA stability.

We next looked at the local secondary structure in the ORF. To do so, we calculated the folding energy within a 100-base sliding window and took the lowest value for each coding region. For both endogenous and ORFeome mRNAs, we found no general relationship between transcript stability and secondary structure (endogenous *r*_*s*_: −0.10 to 0.04 versus ORFeome *r*_*s*_: −0.06 to 0.07, Supplemental Fig. S3C,D). Although it is possible that strong secondary structures influence stabilities for only some transcripts, it appears unable to provide a general explanation for the range of stabilities seen with the ORFeome mRNAs.

We next examined the potential of coding regions to modulate mRNA stability through 3′UTR-like regulation. We first divided ORFeome mRNAs into those containing or lacking sites for the top five expressed microRNA (miRNA) families (Nam et al. 2014). Consistent with individual miRNA sites in the ORF having little impact (Grimson et al. 2007; Schnall-Levin et al. 2011), half-lives for these two sets of ORFeome transcripts failed to be significant for any of the ORFeome data sets (*P* = 0.06 to 0.99, Supplemental Fig. S3E,F). Finally, we compared stabilities for ORFeome mRNAs containing and lacking AU-rich elements, and only observed modest differences, although these were significant for two of the four ORFeome data sets (*P* = 10^−6^ to 0.9, Supplemental Fig. S3G,H). Thus, although many coding regions contain sites that have the potential to be recognized by 3′UTR regulatory factors, these sites cannot explain the range of ORFeome half-lives.

This conclusion is consistent with previous reports showing that miRNA sites in the ORF are rarely effective, because of the translating ribosome (Grimson et al. 2007; Gu et al. 2009). This result also suggests that some of the residual variation in ORFeome mRNA stability upon 4EGI-1 treatment (Fig. 2F) may be due to RBP-based regulation that now has an opportunity to impact stability.

### Codon use is associated with mRNA stability

We next turned to the role of codon use in mRNA stability. To do so, we used a previously defined metric (Presnyak et al. 2015), the codon stability coefficient or CSC. For each nonstop codon, we calculated its frequency in each ORF and measured the Spearman correlation between the codon frequency with the associated mRNA half-life across the collection of endogenous or ORFeome mRNAs (giving rise to endogenous and ORFeome CSC values, respectively). We calculated these scores from each of our three ORFeome/endogenous stability data sets, as well as those generated during DMSO treatment (Fig. 2), giving us four independent measurements for each CSC value.

**FIGURE 3.**
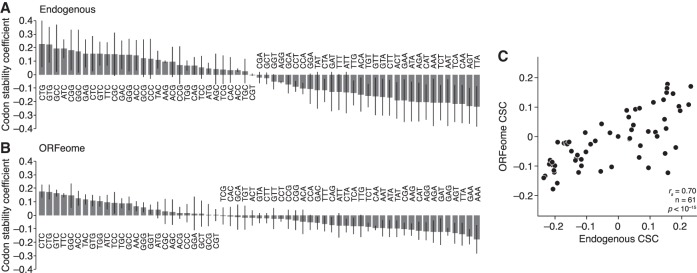
Codon use corresponds to mRNA stability. (*A*) Codons are differentially associated with stability. Shown are average Spearman correlations, for each nonstop codon, of their frequency with mRNA stability (codon stability coefficient; CSC) for endogenous HEK293T mRNAs. The line corresponds to a standard deviation as calculated from the four measurements. (*B*) As in *A*, except for ORFeome mRNAs. (*C*) Endogenous and ORFeome mRNAs have similar CSCs. Plotted are the average CSC values for endogenous mRNAs compared to ORFeome mRNAs.

We calculated the average score of the four values for each codon (Fig. 3A,B; Supplemental Table S5). Overall, the average endogenous and ORFeome CSC values correlated with each other (*r*_*s*_ = 0.70, *P* < 10^−15^; Fig. 3C). Consistent with these effects being mediated by translation, ORFeome values calculated based on computationally +1 or +2 frameshifted codons were significantly less correlated with endogenous CSC values (*r*_*s*_ = 0.32 and 0.22, respectively; *P* < 0.0002, Fishers r-to-z transformation). Similarly, there was not a significant correlation between the ORFeome values and those from yeast, an unrelated species (*r*_*s*_ = 0.22, *P* = 0.07).

In investigating the effects of codons on mRNA stability, much of the focus has been on the effects of synonymous codons because of the model that the abundance of cognate tRNAs differs, leading to differences in decoding of synonymous codons and thus mRNA stability. However, we noted that, surprisingly, some synonymous codons tended to have similar relationships with stability, especially for the “unstable” ORFeome CSCs (Supplemental Fig. S4A). To investigate this issue more thoroughly, as has been done before (Bazzini et al. 2016; Wu et al. 2019b), we calculated an “amino acid stabilization coefficient” (AASC) for the 20 standard amino acids by averaging the CSCs for the corresponding codons (Supplemental Fig. S4B; Supplemental Table S6). As with CSCs, average endogenous and ORFeome AASCs were correlated (*r*_*s*_ = 0.60, *P* = 0.007). Although codons for several amino acids, such as leucine, showed a wide range in stability scores, codons for other amino acids (such as lysine and glutamate) had similar values. Taken together, these data demonstrate that codon usage and mRNA stability are related in human cells, as has been seen in other organisms, and that, for some amino acids, synonymous codons appear to behave similarly.

### Instability-associated codons are translated more slowly

**FIGURE 4.**
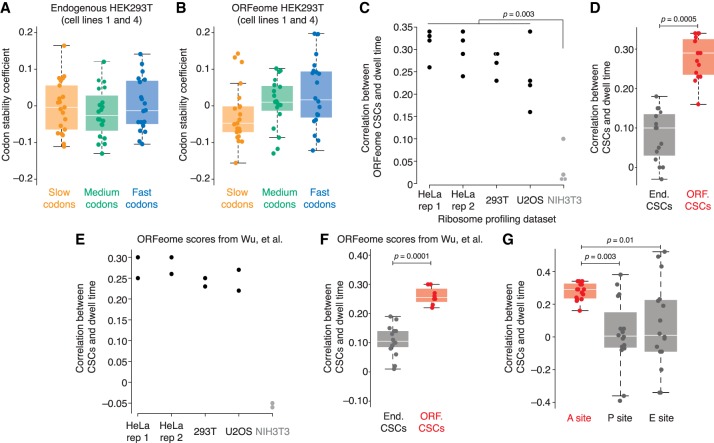
Codons associated with instability are translated more slowly. (*A*) Endogenous HEK293T CSCs weakly correspond with pause scores. Using HeLa ribosome profiling (Arango et al. 2018), elongation speeds were calculated for each codon in the A site, and then codons were divided into three groups (slow in orange; neutral in green; fast in blue). Shown are boxplots and points for the corresponding CSC values as determined by endogenous HEK293T mRNAs. (*B*) As in *A*, except for ORFeome-derived CSCs. (*C*) Comparison of ORFeome CSCs and elongation speeds. Four independent sets of ORFeome CSC values were compared with elongation speeds derived from five different ribosome profiling experiments (Eichhorn et al. 2014; Arango et al. 2018). Plotted are the resulting Spearman correlations for each of the 20 comparisons with those including NIH3T3 elongation speeds shown in gray. Significance was determined by the Wilcoxon test. (*D*) ORFeome stability scores better correlate with elongation speeds than endogenous scores. Similar to *C*, four independent sets of CSC values derived from matched endogenous and ORFeome mRNAs were compared with the four values of human elongation speeds (from HeLa rep 1, HeLa rep 2, HEK293T, and U2OS ribosome profiling experiments). Shown are boxplots and points for the Spearman correlations from the 16 resulting comparisons for endogenous (gray) and ORFeome (red) CSC values. Significance was determined by the Wilcoxon test. (*E*) Published ORFeome CSC values derived in HEK293T and K562 cells (Wu et al. 2019b) were compared with elongation speeds determined from ribosome profiling in HeLa, HEK293T, U2OS, and NIH3T3 cells. Plotted are the corresponding correlations for each of the 10 comparisons; comparisons with NIH3T3 scores are shown in gray. Significance was determined by the Wilcoxon test. (*F*) Published ORFeome CSCs correspond better to elongation speeds than endogenous CSCs. Spearman correlations between each of the human-line elongation speeds and the published endogenous and ORFeome CSC values (Wu et al. 2019b) were calculated. Correlations with endogenous CSCs are shown in gray; with ORFeome CSCS, in red. Significance was determined by the Wilcoxon test. (*G*) A-site speeds correspond better to stability scores than P- and E-site scores. P- and E-site scores were calculated for the four ribosome profiling data sets from human cells (HeLa rep 1, HeLa rep 2, HEK293T, and U2OS), and compared with the ORFeome CSC values. Plotted are the Spearman correlations for the 16 comparisons for A-site scores (in red), P- and E- site scores (in gray). Significance was determined by the Wilcoxon test.

Based on studies in model organisms, the current model explaining the relationship between codon usage and mRNA stability is that some codons are translated slowly, and slow elongation in turn triggers mRNA degradation. We thus asked whether this model held in human cells by investigating the relationship between stability coefficients and elongation rates. To do so, we used a published ribosome profiling data set from HeLa cells (Arango et al. 2018) and calculated the enrichment of each codon (when in the inferred A site) in ribosome-protected fragments compared with its background abundance in the pool of translated transcripts (see Materials and Methods, Supplemental Table S5). That is, high enrichment indicates long dwell times and thus slow relative elongation; low enrichment, short dwell times and fast relative elongation. As expected, we observed a strong enrichment for stop codons in the A site, and depletion in the P site (data not shown). We then compared these relative dwell times with the endogenous and ORFeome CSCs. When we examined the codon scores from cell lines 1 and 4, there was no relationship between endogenous CSCs and dwell times (*r*_*s*_ = 0.02, *P* = 0.83), but there was a significant correlation with the ORFeome values (*r*_*s*_ = 0.33, *P* = 0.008) such that codons translated more slowly were associated with instability (Fig. 4A,B).

To explore the relationship between codon stability measurements and elongation speeds more fully, we calculated codon-level dwell times from four other ribosome profiling data sets: a biological replicate from HeLa cells (Arango et al. 2018); HEK293T cells (Eichhorn et al. 2014); U2OS cells (Eichhorn et al. 2014); and NIH3T3 cells (Eichhorn et al. 2014). We next compared our four sets of ORFeome CSCs with the calculated dwell times from both HeLa replicates, as well as HEK293T, U2OS, and NIH3T3 experiments, given 20 total comparisons (Fig. 4C). Consistent with the known differences in codon usage and tRNA abundance between mouse and human cells, the ORFeome CSCs were significantly better correlated with human ribosome speeds than NIH3T3 ribosome speeds (human line *r*_*s*_ values: 0.16 to 0.34; NIH3T3 *r*_*s*_ values: 0.01 to 0.10; *P* = 0.003, Wilcoxon test; Fig. 4C). Importantly, when we repeated this analysis with the corresponding endogenous CSC values, the ORFeome values were significantly better correlated with human ribosome dwell times than the endogenous values (*P* = 0.0005, paired Wilcoxon test; Fig. 4D).

We next compared the ORFeome CSC values determined in the contemporaneous study (Wu et al. 2019b) with A-site dwell times. As with our ORFeome values, these ORFeome CSCs were significantly more correlated with human ribosome dwell times than those in NIH3T3 cells (*P* = 0.04, Wilcoxon test; Fig. 4E). Similarly, their ORFeome CSCs performed significantly better than the corresponding endogenous values (*P* = 0.0001, Wilcoxon test; Fig. 4F). Interestingly, despite their CSC values being derived from many more ORFs than our values, there was no difference in the correlations of either set with ribosome elongation speeds (*P* = 1, Wilcoxon test). Taken together, these results indicate that codons associated with instability—but only when determined by ORFeome, not endogenous, measurements—are translated more slowly.

### ORF-regulation of mRNA stability predominantly relates to codons in the A site

Although the A-site codon is thought to predominantly influence elongation speeds, other steps in elongation, such as peptide bond formation or translocation, can also influence ribosome speed and so could conceivably also impact mRNA stability. To explore this possibility, we calculated dwell times as before, but this time for codons in the inferred P and E sites, and compared the four sets of human dwell times for codons in the A, P, and E sites with our four sets of ORFeome CSC measurements.

Although correlations between CSCs and A-site speeds were tightly clustered, there was a broader range with the P- and E-site speeds (Fig. 4G). Overall, however, the effect size of the correlations was negligible for P- and E-site speeds (median correlation: 0.29, 0.01, and 0.01 for A, P, and E sites, respectively), and A-site speeds correlated significantly better with ORFeome CSC values than P- or E-site speeds (*P* = 0.003 and 0.01, respectively, paired Wilcoxon test). These results suggest that the A-site speed is likely the major contributor to ORF-mediated regulation of mRNA stability.

## DISCUSSION

Inspired by reports of coding regions affecting mRNA stability in model organisms, we set out to investigate this phenomenon in human cells. By using the ORFeome collection, where human coding regions are surrounded by invariant UTRs, we showed that ORFs impact mRNA stability in human cells and that the majority of the effect depends on translation. Through our ORFeome stability measurements, we were able to explore features that affect stability, and, in contrast to previous suggestions (Duan et al. 2013; Geisberg et al. 2014; Neymotin et al. 2016), we found little evidence that ORF length, local secondary structure, and 3′UTR-like regulation generally impact mRNA stability. Some features (e.g., miRNA-mediated regulation) likely regulate specific mRNAs; but others (e.g., length) may coevolve with direct effectors of RNA stability, explaining results from studies that relied on measurements of endogenous transcript stability.

Instead, coding regions seem to affect mRNA stability through differences in codon usage. Based on our results, experiments in model organisms (Presnyak et al. 2015; Bazzini et al. 2016; Boël et al. 2016; Mishima and Tomari 2016; Mattijssen et al. 2017; de Freitas Nascimento et al. 2018; Jeacock et al. 2018; Wu et al. 2019b), and an independent study in human cells (Wu et al. 2019b), we propose that the relationship between codon use and mRNA stability is causative, and that instability codons stimulate mRNA decay by slowing translation elongation.

One of the strongest lines of evidence for the connection between mRNA stability and translation elongation speeds comes from a comparison of codon stability scores from ORFeome and endogenous mRNAs with elongation speeds. While A-site dwell times did not correlate with endogenous scores (which derive from a mix of ORF- and UTR-based regulation), we saw a reproducible, significant correlation with ORFeome stability scores (which derive from only ORF-based regulation).

Because we found the strongest association between instability-associated codons and amino acids with A-site speeds, we favor a model that the elongation speeds (and thus mRNA stability) are predominantly determined by decoding rates, although there may be some amino acids (such as proline) that slow elongation by other mechanisms (such as peptide bond formation) (Lu and Deutsch 2008; Wohlgemuth et al. 2008; Charneski and Hurst 2013; Gutierrez et al. 2013; Artieri and Fraser 2014; Gardin et al. 2014). In turn, decoding rates are probably determined by a combination of tRNA abundance and aminoacylation. Interestingly, we found that synonymous codons tend to have similar A-site dwell times (Supplemental Fig. S4B), and such “amino acid” effects have now been seen in several studies in mammalian cells (Darnell et al. 2018; Gobet et al. 2019; Wu et al. 2019a). Moreover, we found that A-site dwell times for different amino acids differ between HeLa/HEK293T and U2OS cells (Supplemental Fig. S4C,D). Thus, we propose that the combination of tRNA abundance and charging affect decoding rates and mRNA stability in human cells.

Nonetheless, an unanswered question is why the codon stability scores do not correlate better with A-site dwell times. There are several possible technical and biological explanations. For instance, this result may be a technical artifact of ribosome profiling because it is known that ribosomes can carry out one or two rounds of elongation during the polysome preparation, which could blur the resolution of elongation speeds. Arguing against this possibility is that the P site and A site elongation speeds are very different. Similarly, it is unlikely that this result is due to only using a few hundred transcripts for the ORFeome scores: Corresponding scores from a parallel paper (Wu et al. 2019b), which were based on thousands of transcripts, did not perform any better.

Instead, we suspect there may be several biological explanations. First, within endogenous coding regions, there is a co-usage of codons and amino acids, which may partially confound the stability scores for individual codons and amino acids. Second, in yeast, di- and tricodons have been shown to dramatically affect protein output (Gamble et al. 2016). Such multicodon effects are not captured by either our stability scores or current elongation speed measurements. Finally, although our data indicate that coding regions primarily affect mRNA stability through translation, translation-independent effects remain unaccounted for in our current analysis and could explain some of the stability variation observed in the ORFeome.

One final issue remains: What is the overall contribution of the ORF to mRNA stability? Although our work and that from others indicate that coding regions impact mRNA stability, the amount to which they do so remains unclear. Based on what we have already found, determining the overall contribution of the coding region to mRNA stability will be a challenge, and the answer probably differs for different transcripts and between different cell types. That is, because the effect of a coding region depends upon ribosome loading, endogenous mRNAs, which have different translation initiation rates, will differ in their opportunity to be regulated by elongation speed. Indeed, it is likely that those mRNAs most affected are those both with high initiation rates and containing destabilizing codons. Another important consideration is that tRNA abundance and charging change between different cell states (Dittmar et al. 2006; Goodarzi et al. 2016; Darnell et al. 2018; Torrent et al. 2018), and so the identities of mRNAs with destabilizing coding regions may also change between different cell types. One important wrinkle is the potential for interplay between ORF and 3′UTR regulation. For instance, a recent report indicated that, in *Drosophila* S2 cells, the coding region can influence the amount of regulation exerted by 3′UTR sites (Cottrell et al. 2017), but it is unknown whether the same interaction occurs in human cells. Although integrating the contributions of different parts of an mRNA on stability requires additional work, it is the critical next step for understanding how gene expression is controlled.

## MATERIALS AND METHODS

### Cell lines and growth conditions

#### Human cell lines

Human HEK293T cells were cultured in DMEM (Lonza) supplemented with 10% FBS (VWR Seradigm) and 1% penicillin–streptomycin solution. Cell lines were cultured at 37°C in a humidified incubator with 5% CO_2_.

#### Drosophila cell lines

*Drosophila melanogaster* Schneider 2 (S2) cells (Cat #R69007, Thermo Fisher Scientific) were cultured in ExpressFive SFM media (Thermo Fisher Scientific) supplemented with 10% heat-inactivated FBS (Wisent) and 20mM l-Glutamine (Thermo Fisher Scientific) at 28°C.

#### Yeast strains

*S. cerevisiae* USY006 was grown in YPD liquid or plates at 30°C. Cultures were obtained from Dr. John Rubinstein (The Hospital for Sick Children).

### ORFeome cell line preparation

The human ORFeome collection version 8.1 (ccsbBroad304) cloned into lentiviral vector pLX304 was obtained from Dr. Jason Moffat (University of Toronto) as a series of 96-well overnight bacterial cultures. Equal volumes of bacterial cultures were pooled into 36 pools comprising ∼576 clones each. Plasmid DNA was isolated using the GeneJET Plasmid Midiprep Kit (Thermo Fisher Scientific) as per manufacturer's instructions, yielding an average of ∼70 µg plasmid DNA per pool. The 36 isolated pools were further combined into six unique pools for downstream cell line generation.

Each of the six unique virus pools were packaged by transfection into HEK293T cells using Lipofectamine 2000 (Thermo Fisher Scientific), according to manufacturer's instruction. Cells were transfected with the lentivirus pLX304 pool, psPAX2 packaging vector, and pVSV-G envelope vector. After 8 h, transfection media was then removed and switched to harvest media (DMEM + 10% FBS + 1.1 g/100 mL BSA [7.5% solution, Thermo Fisher Scientific]). Cells were left for 2 d to complete virus production. Media was then collected from the plate and filtered through a 0.45 µm filter by syringe. Harvested viruses were aliquoted.

Freshly thawed HEK293T cells were grown in 10 cm dishes to reach ∼30%–50% confluence for the day of infection. Media was removed and 9 mL prewarmed infection media (DMEM + 10% FBS + 8 µg/mL Polybrene) was added to cells. A total of 2 mL of freshly harvested virus pool was added to one plate of HEK293T cells each and incubated overnight. Cells were then trypsinized and expanded into 15 cm dishes. Cells were selected using selection media (DMEM + 10% FBS + 6 µg/mL Blasticidin [BioShop]) for 6 d. Selection media was changed every day. Cells were then frozen in cell freezing medium (Sigma-Aldrich) and stored in liquid nitrogen.

### Western blotting

Cells were harvested by trypsinization and pelleted by centrifugation at 1000*g* for 2 min at 4°C. Cell pellets were resuspended in 500 µL lysis buffer (100 mM KCl, 0.1 mM EDTA, 20 mM HEPES-KOH pH 7.6, 0.4% NP-40, 10% glycerol, 1 mM DTT, complete mini EDTA-free protease inhibitors [Roche]) and clarified at 21,000*g* for 5 min at 4°C. A total of 250 µL supernatant was mixed with 20 µL 4× Bolt LDS sample buffer (Thermo Fisher Scientific), 8 µL 10× Bolt sample reducing agent (Invitrogen) and proteins were denatured at 75°C for 10 min. Protein samples were loaded into Bolt 4%–12% Bis-TRIS Plus gels (Thermo Fisher Scientific) and run at 160 V for ∼1 h. The gel was transferred onto an Amersham Hybond PVDF membrane (GE Healthcare), according to manufacturer's instructions. Primary antibodies were added at 1:10,000 concentration for α-V5 antibodies (Cat #V8012, Sigma-Aldrich), 1: 10,000 for α-puromycin antibodies (Cat #3RH11, Cedarlane), and 1: 5000 for α-tubulin antibodies (Cat #T5168 Sigma-Aldrich). α-mouse secondary antibody (Cat #7076, New England Biolabs) was used at 1:10,000. Blots were imaged using ECL Prime Western Blotting Detection Reagent (GE Healthcare) and exposed on Amersham Hyperfilm (GE Healthcare).

### Polysome fractionation

hORF cell line 1 was grown for 24 h in the presence of either DMSO or 100 µM 4EGI-1 (Cedarlane). Cells were treated with 100 µg/mL cycloheximide (CHX) (BioShop) for 10 min. Cells were harvested on ice by washing 2× with ice-cold PBS containing 100 µg/mL CHX, and lysing with 500 µL ice-cold filter-sterilized lysis buffer (10 mM Tris-HCl [pH 7.4], 5 mM MgCl_2_, 100 mM KCl, 1% Triton X-100, 2 mM DTT, 500 U/mL RNasin [Promega], 100 µg/mL CHX, Protease inhibitor [1× complete, EDTA-free, Roche]). Cells were scraped off the dish into tubes and sheared gently 4× with a 26-gauge needle. Lysed cells were centrifuged at 1300*g* for 10 min at 4°C, and clarified supernatant was isolated.

A 10/50% sucrose gradient was created by combining heavy and light solutions on a BioComp Gradient Master. Heavy and light solutions consisted of 20 mM HEPES-KOH (pH 7.4), 5 mM MgCl_2_, 100 mM KCl, 2 mM DTT, 100 µg/mL CHX, and 20 U/mL SUPERaseIn, and either 10% or 50% sucrose (w/v), respectively. An amount of 300 µL of samples was layered on sucrose gradients and centrifuged in a precooled Beckman Ultracentrifuge L-90K using SW41 rotor at 36,000 RPM (221632.5G) for 2 h at 4°C. The gradient was fractionated using the BioComp Piston Gradient Fractionator and absorbance measurements were made using an Econo EM-1 UV Monitor (BioRad).

### Puromycin incorporation assay

hORF cell line 1 was grown for 1, 8, or 24 h in the presence of either DMSO, 100 μM 4EGI-1, or 5 μg/mL cycloheximide. Cells were pulsed with 1.5 μg/mL puromycin dihydrochloride (Thermo Fisher Scientific) for 10 min at 37°C. Cells were then harvested and lysed as above.

### HEK293T endogenous and ORFeome mRNA stability determination by metabolic labeling

#### Generation of spike-in RNA

Two sets of spike-in RNA were generated. An unlabeled *S. cerevisiae* spike-in is used to determine the enrichment of 4SU-labeled RNA over unlabeled RNA, as described previously (Lugowski et al. 2017). *S. cerevisiae* strain USY006 was grown in YPD liquid culture at 30°C, and RNA was isolated using a hot acidic phenol method (Rissland and Norbury 2009). A 4SU-labeled *D. melanogaster* spike-in was also generated by supplementing S2 culture media with 100 µM 4SU for 24 h prior to harvesting. RNA was extracted using TRI-reagent (Molecular Research Center) as per manufacturer's instructions.

#### Metabolic labeling of hORFeome cell lines

Freshly thawed HEK293T hORFeome cell lines were cultured for three to four passages and seeded into DMEM + FBS culture media in 15 cm dishes such that they attained ∼50% confluence on the day of the time course. Media was replaced with DMEM + 10% FBS + 100 µM 4SU (Sigma-Aldrich) reconstituted in DMSO. Cells were harvested at 1, 2, 4, 8, 12, and 24 h after addition of 4SU. Harvesting was performed by dislodging cells off the plate during two washes with cold 1× PBS followed by spinning at 1000*g* for 5 min at 4°C. Cell pellets were resuspended in 1 mL TRI-Reagent (Molecular Research Center) and extracted according to manufacturer instructions.

For translation inhibition experiments, hORF cell line 1 or cell line 4 cells growing in 10 mL DMEM + FBS in 10 cm dishes were pretreated with either 0.1% DMSO or 100 µM 4EGI-1 (Cedarlane) dissolved in DMSO for 1 h. Following this, 100 µM 4SU was added to media for all plates, and the time course was performed as described above.

#### Reversible biotinylation and fractionation of 4SU-labeled mRNAs

RNA was labeled as described previously (Lugowski et al. 2017). Briefly, 100 µg of total hORF RNA was mixed with 10 µg unlabeled *S. cerevisiae* RNA (i.e., 10% w/w) and 10 µg 4SU-labeled S2 *D. melanogaster* RNA (i.e., 10% w/w). Water was added to bring the volume up to 120 µL. An amount of 1 mg/mL HPDP-biotin (Thermo Fisher Scientific) was reconstituted in dimethylformamide by shaking at 37°C for 30 min at 300 RPM. 120 µL of 2.5× Citrate buffer (25 mM citrate, pH 4.5, 2.5 mL EDTA) and 60 µL of 1 mg/mL HPDP-biotin were added to the premixed RNA sample for each time point. This solution was incubated at 37°C for 2 h at 300 RPM on an Eppendorf ThermoMixer F1.5 in the dark. Samples were extracted twice with acid phenol, pH 4.5 (Invitrogen), and once with chloroform. RNA was precipitated with 18 µL 5M NaCl, 750 µL 100% ethanol, and 2 µL GlycoBlue (Invitrogen) overnight at −20°C. Precipitated RNA was pelleted for 30 min at 21,000*g* at 4°C. The RNA pellet was resuspended in 200 µL of 1× wash buffer (10 mM Tris-HCl, pH 7.4, 50 mM NaCl, 1 mM EDTA).

Biotinylated RNA was purified using the µMACS Streptavidin microbeads system (Miltenyi Biotec). An amount of 50 µL Miltenyi beads per sample were preblocked with 48 µL 1× wash buffer and 2 µL yeast tRNA (Invitrogen), rotating for 20 min at room temperature. µMACS microcolumns were washed 1× with 100 µL nucleic acid equilibration buffer (Miltenyi Biotec), followed by 5× washes with 100 µL 1× wash buffer. Beads were applied to microcolumns in 100 µL aliquots, and again washed 5× with 100 µL 1× wash buffer. Beads were demagnetized and eluted off the column with 2× 100 µL 1× wash buffer, and columns were placed back on the magnetic stand. A total of 200 µL beads was mixed with each sample of biotinylated RNA and rotated at room temperature for 20 min.

Samples were then applied to the microcolumns in 100 µL aliquots, washed 3× with 400 µL wash A buffer (10 mM Tris-HCl, pH 7.4, 6 M urea, 10 mM EDTA) prewarmed to 65°C, and then washed 3× with 400 µL wash B buffer (10 mM Tris-HCl, pH 7.4, 1 M NaCl, 10 mM EDTA). RNA was eluted with 5× 100 µL of 1× wash buffer supplemented with 0.1 M DTT, and flow through was collected in a tube. Purified RNA was precipitated with 30 µL 5 M NaCl, 2 µL GlycoBlue, and 1 mL 100% ethanol, incubated at −20°C overnight. Samples were then spun at 21,000*g* at 4°C for 30 min and resuspended in 20 µL water. RNA quality was assessed by running 3 µL of samples on a ∼1.5% agarose gel.

#### Generation of next-generation sequencing libraries and RNA-sequencing

A total of 10 µL of purified 4SU-labeled RNA or unpurified total RNA from the 24-h time point was used to prepare RNA-seq libraries using the TruSeq Stranded mRNA Sample Preparation Kit (Illumina), according to manufacturer's instructions. Adapter-ligated fragments were enriched with 14× PCR cycles. Approximately 16 to 22 samples were multiplexed on a single lane in an Illumina HiSeq 2500 at The Centre for Applied Genomics (The Hospital for Sick Children, University of Toronto) to obtain ∼10 million 50 bp single-end reads per sample. The data is available from GEO, accession GSE123165.

### HEK293T endogenous and ORFeome mRNA half-life calculations

#### Reference genome information

Human (hg38), *D. melanogaster* (dm6), and *S. cerevisiae* (sacCer3) genomes were obtained in 2bit format using the UCSC Table Browser (Karolchik et al. 2004). 2bit files were converted to FASTA using the kentUtils command twoBitToFa, and GTF annotations were downloaded using the kentUtils command genePredToGtf. The three genomes were combined using custom bash scripts to make a hg38 + dm6 + sacCer3 genome.

#### Initial processing of sequencing reads

Library quality was assessed using FastQC v0.11.5 (http://www.bioinformatics.babraham.ac.uk/projects/fastqc). Reads were trimmed and clipped for Illumina adapters using Trimmomatic v0.36 (Bolger et al. 2014) using the following settings: -phred33 ILLUMINACLIP: TruSeq3-SE.fa:2:30:10 LEADING:3 TRAILING:3 SLIDINGWINDOW:4:15 MINLEN:36.

#### Genome mapping and counting

Trimmed reads were aligned to the indexed hg38 + dm6 + sacCer3 genome using STAR version 2.5.2 (Dobin et al. 2013) with the following nondefault settings: –outFilterMultimapNmax 10 –outFilterMismatchNoverLmax 0.05 –outFilterScoreMinOverLread 0.75 –outFilterMatchNminOverLread 0.85 –alignIntronMax 1 –outFilterIntronMotifs RemoveNoncanonical –outSAMtype BAM SortedByCoordinate –quantMode GeneCounts.

HTSeq version 0.6.1 (Anders et al. 2015) was then used to quantify gene counts from aligned BAM files using the following settings: –order=pos –stranded=reverse –minaqual=10 –mode=intersection-strict. Note that counting of features from human CDS and intronic GTF files were performed separately.

#### Defining hORF genes

Gene counts were loaded into RStudio version 3.3.1. Dplyr package (https://CRAN.R-project.org/package=dplyrnn) was used for all data manipulation and filtering. Steady state RNA-sequencing counts mapping to human CDS features were obtained from each cell line and normalized to library size to allow comparisons across samples. For a given cell line X, genes were described as “detectable hORF genes” if they met each of the following conditions:

They were in the list of hORFs infected into cell line X;
Normalized steady state RNA-sequencing for cell line X was greater than threefold that in the matched cell line;
Normalized steady state RNA-sequencing for cell line X was greater than four reads.

Genes that were not infected into cell line X were described as endogenous genes.

#### Calculation of mRNA half-lives

All half-life calculations were performed in RStudio version 3.3.1 as described previously (Lugowski et al. 2018). Briefly, read counts for mature human mRNAs were filtered such that each gene had at least one read mapped to its CDS at each time point, and at least five reads mapped at any (at least one of six) time point. CDS-mapping reads for each gene at each time point were then normalized to the sum of all corresponding *D. melanogaster* mapping reads.

Half-lives were calculated by fitting these normalized read counts at each time point to a bounded growth equation using weighted nonlinear least squares. The bounded growth equation has been previously described (Lugowski et al. 2017). Briefly, the equation states:

$$y(t)=y_{eq}\times (1-e^{kt}),$$

where *y*(t) is the amount of a given transcript remaining at time *t*, *y*_eq_ is the amount of that transcript at steady state, and *k* is the transcript-specific decay constant.

The nls() function in the stats package was used to fit the time points to the equation above, with settings equivalent to the following:

- start = c(*y*_eq_ = max(y), *k* = −0.5)
- algorithm = “port”
- weights = 1/*y*(t)
- lower = c(*y*_eq_ = 0, *k* = −Inf), upper = c(*y*_eq_ = Inf, *k* = 0)

If the data did not converge, a value of NA was returned. The half-life of each transcript is then obtained using the following equation:

$$HL=\frac{ln(2)}{k}$$

### Calculation of local secondary structure

To measure local secondary structure, each gene's coding sequence was assayed by sliding 100 bp windows, each starting 3 bp apart. Each window was folded using ViennaRNA version 2.2.8 RNAfold function (Lorenz et al. 2011) using default parameters. Minimum folding energy (MFE) for each 100 bp sequence was extracted from output files using custom bash scripts. Median and minimum MFEs across each CDS were determined using group_by and summarize functions in the dplyr package in RStudio.

### Codon and amino acid stability coefficient calculations

Codon usage frequency was calculated from each gene's coding sequence using the seqinr package's uco function. For frame shift controls, codon and amino acid usage were calculated after shifting the frame by +1 (removing positions 1, *n* − 2, and *n* − 1 from CDS of length *n*) and +2 (removing positions 1, 2, and *n* − 1 from CDS of length n).

As described previously (Radhakrishnan et al. 2016), codon stability coefficients (CSC) from a given half-life data set were calculated by determining the Spearman correlation between the codon frequency for each codon in a transcript with the measured half-lives of that transcript. Stop codons were excluded from CSC calculations. AASCs were calculated by taking the arithmetic mean of the CSC values for the corresponding codon(s).

### Codon and amino acid-specific pause score calculations using Ribo-Seq

Ribosome profiling data sets for HeLa cells were obtained from GEO, accession GSE102113, and for HEK293T, U2OS, and NIH3T3 cells from GEO, accession GSE83616. To identify codon enrichments in the A, P, and E sites of translating ribosomes from ribosome profiling data, reads were first trimmed of their adapters using cutadapt v2.1 (Bolger et al. 2014). Trimmed reads were then aligned to the human genome (hg38, Gencode version 28) or the mouse genome (mm10, Gencode version 17) using STAR v2.5.2 (Dobin et al. 2013). Only uniquely aligned reads of length 28, 29, or 30 were then used for further analyses. The A, P, and E sites of these reads were defined as positions 17–19, 14–16, and 11–13, respectively. If these sites were not in-frame in a coding sequence, the read was ignored. If they were, the codons in the A, P, and E sites were recorded, allowing the calculation of the frequency with which each codon appeared within each site. This frequency was then compared to the null expected frequency. The null expected frequency for a codon was calculated as its frequency in the longest ORF of each gene weighted by the abundance of the gene in the ribosome profiling data set, using only 28–30 nt, in-frame reads. Amino acid pause scores were calculated by taking the arithmetic mean of the codon scores for the corresponding codon(s).

### Other statistical analyses

Number of replicates, statistical tests used, and *P*-values are specified in the figures and figure legends.

## DATA DEPOSITION

The accession number for the raw data files reported in this paper is GEO GSE123165. The accession number for the HeLa ribosome sequencing data used in this paper is GEO GSE102113. The accession number for the HEK293T, U2OS, and NIH3T3 ribosome sequencing data used in this paper is GEO GSE60426.

## SUPPLEMENTAL MATERIAL

Supplemental material is available for this article.

## Supplementary Material

Supplemental Material
