# Supplementary material for: Coding regions affect mRNA stability in human cells
Source: RNA. 2019 Dec;25(12):1751–64. doi: 10.1261/rna.073239.119 (PMC6859850; doi:10.1261/rna.073239.119)
Supplement: Supplemental Material [file supp_25_12_1751__index.html]

Coding regions affect mRNA stability in human cells — Coding regions affect mRNA stability in human cells — Supplemental Material 

# Coding regions affect mRNA stability in human cells

## Supplemental Material

- Supplemental\_Figure\_1.pdf
- Supplemental\_Figure\_2.pdf
- Supplemental\_Figure\_3.pdf
- Supplemental\_Figure\_4.pdf
- Supplemental\_Figure\_Legends.docx
- Supplemental\_Table\_S1.csv
- Supplemental\_Table\_S2.csv
- Supplemental\_Table\_S3.csv
- Supplemental\_Table\_S4.csv
- Supplemental\_Table\_S5.xlsx
- Supplemental\_Table\_S6.xlsx
