# Supplementary figures and images for: Coding regions affect mRNA stability in human cells
Source: RNA. 2019 Dec;25(12):1751–64. doi: 10.1261/rna.073239.119 (PMC6859850; doi:10.1261/rna.073239.119)

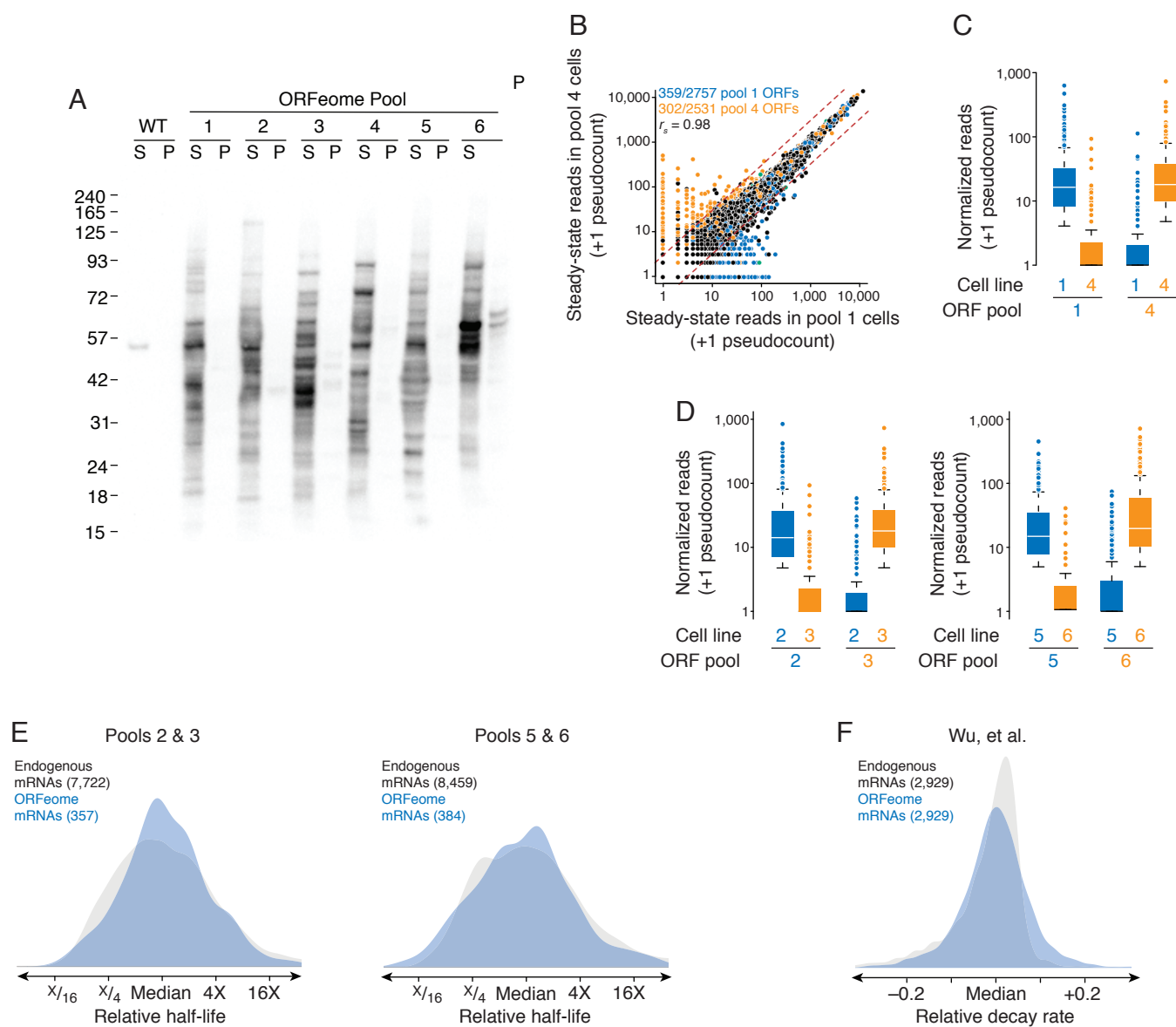

Supplement: Supplemental Material [file supp_073239.119_Supplemental_Figure_1.pdf]

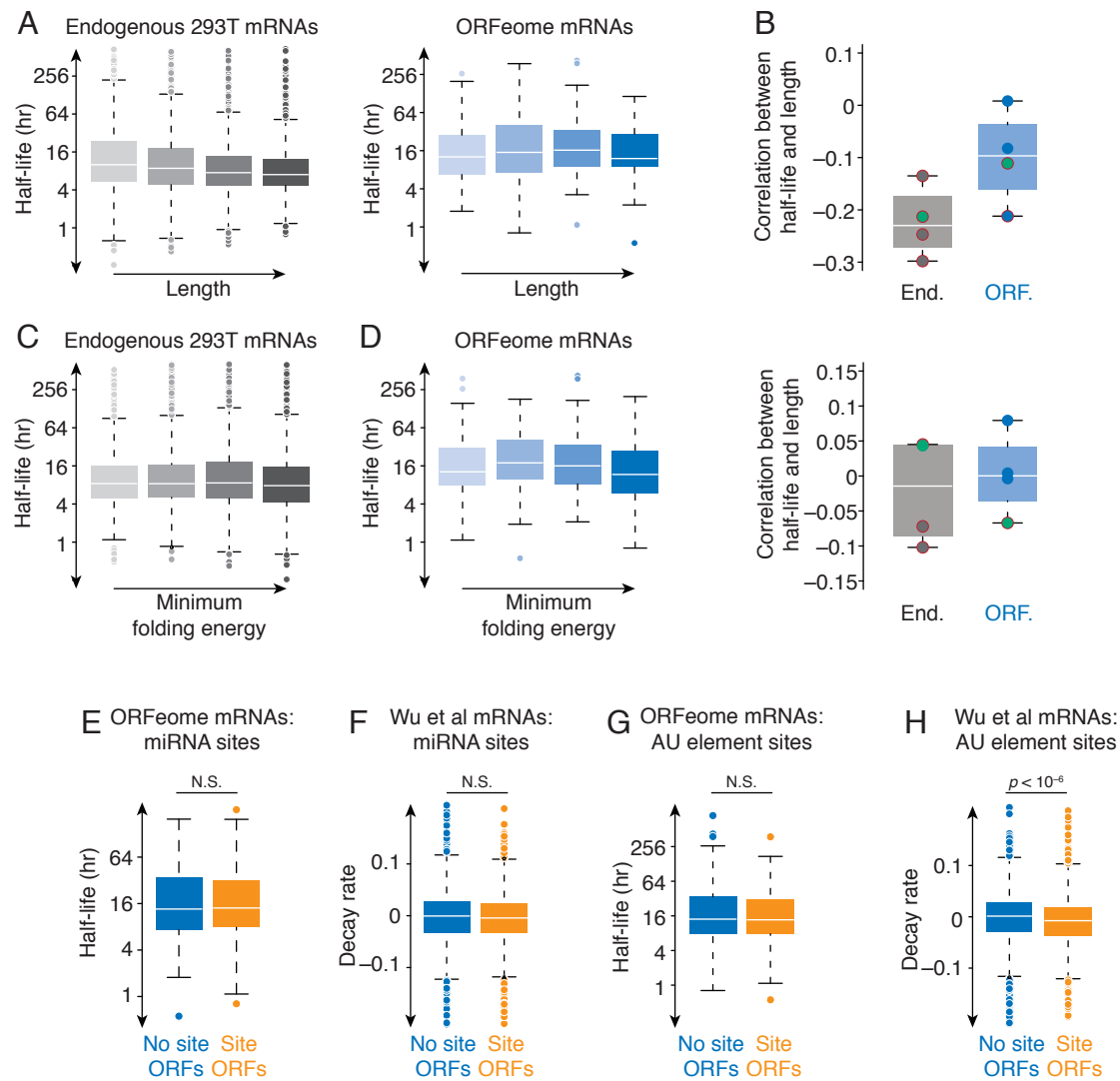

Supplement: Supplemental Material [file supp_073239.119_Supplemental_Figure_3.pdf]

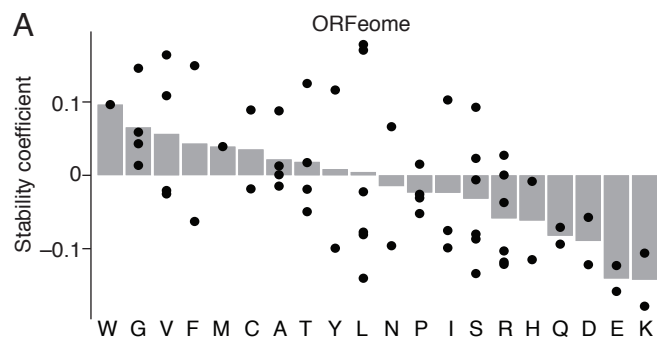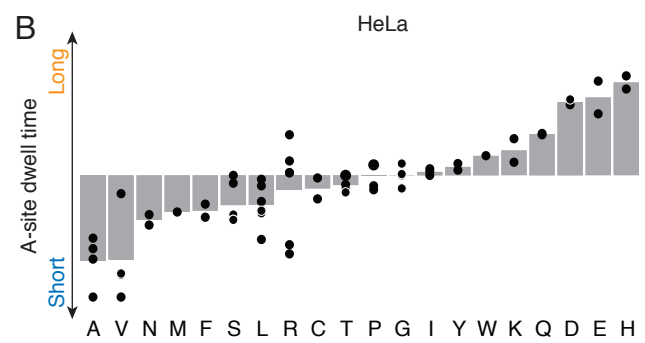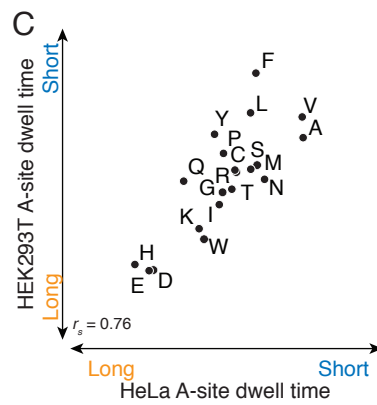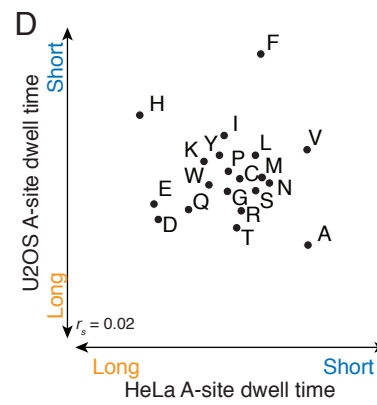

Supplement: Supplemental Material [file supp_073239.119_Supplemental_Figure_4.pdf]
