# Supplementary material for: Coding regions affect mRNA stability in human cells
Source: RNA. 2019 Dec;25(12):1751–64. doi: 10.1261/rna.073239.119 (PMC6859850; doi:10.1261/rna.073239.119)
Supplement: Supplemental Material [file supp_073239.119_Supplemental_Figure_2.pdf]

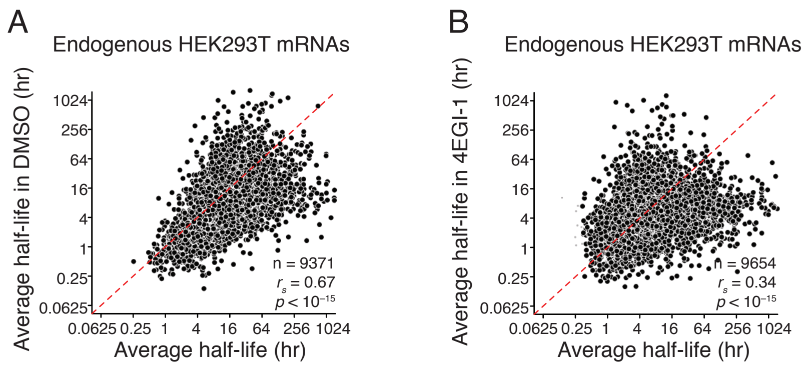

Figure S2. The effect of 4EGI-1 on mRNA stability. (A) DMSO treatment does not substantially affect mRNA stability. Shown are scatterplots comparing half-lives for endogenous genes (averaged from both pools) from the original experiment and DMSO-treated cells. Red dashed line represents x = y. (B) 4EGI-1 treatment affects mRNA stability. As in A, except comparing half-lives from the original experiment and 4EGI-1-treated cells.
