# Supplementary material for: Coding regions affect mRNA stability in human cells
Source: RNA. 2019 Dec;25(12):1751–64. doi: 10.1261/rna.073239.119 (PMC6859850; doi:10.1261/rna.073239.119)
Supplement: Supplemental Material [file supp_073239.119_Supplemental_Figure_Legends.docx]

**SUPPLEMENTAL FIGURE LEGENDS**

**Figure S1. Validation of the ORFeome lines.**

(A) ORFeome complexity was maintained through stable cell line generation. Shown is a western blot probing lysates from the pooled ORFeome stable lines with V5 (the common C-terminal tag in the ORFeome collection). WT, parental HEK293T line; S, supernatant; P, pellet. (B) ORFeome-derived mRNAs are expressed in the stable cell lines. Shown is a scatter plot comparing steady-state RNA-seq reads (with a +1 pseudocount) for each gene between the two pooled lines used in this study. In black, genes in neither pool; in blue, genes in pool 1; in orange, genes in pool 4; in green, genes in both pools. Red dashed lines represent y = 3X and y = X/3, which were used as cut-offs to classify genes as ORFeome-expressed. ORFeome genes that did not pass threshold were not used for subsequent analysis (see Methods for more details). Numbers refer to the total number of genes in each pool and the number passing the 3-fold threshold. (C) ORFeome mRNAs are expressed in a pool-dependent fashion. Shown are boxplots of normalized read counts (with a +1 pseudocount) for ORFeome-derived mRNAs (split into pool 1 and pool 4) in the two pooled stable cell lines. Abundance in cell line 1 is shown in blue; in cell line 4, in orange. Note that the ORFeome pools are expressed in the appropriate cell line. (D) As in (C), except for cell lines 2 and 3 (left) and 5 and 6 (right). (E) ORFeome mRNAs show as much variability in stability as endogenous mRNAs. Plotted are the density distributions of median-centered stabilities of endogenous and ORFeome mRNAs (in grey and blue, respectively) for cell lines 2 and 3 (left) and 5 and 6 (right). (F) As in (E), except for matched endogenous and ORFeome transcripts from Q. Wu, et al. 2019.

**Figure S2. The effect of 4EGI-1 on mRNA stability.**

(A) DMSO treatment does not substantially affect mRNA stability. Shown are scatterplots comparing half-lives for endogenous genes (averaged from both pools) from the original experiment and DMSO-treated cells. Red dashed line represents x = y. (B) 4EGI-1 treatment affects mRNA stability. As in A, except comparing half-lives from the original experiment and 4EGI-1-treated cells.

**Figure S3. Known modulators of mRNA stability cannot explain the effects mediated by the coding region.**

(A) Correlation between mRNA stability and ORF length. Shown are boxplots for half-lives of endogenous HEK293T and ORFeome mRNAs binned into the same quartiles by ORF length (left and right, respectively). (B) ORFeome mRNA stability does not correlate with length. Plotted are the correlations between the four endogenous mRNA stability measurements and length (left), and the four ORFeome mRNA stability measurements (right). Those correlations derived from the Q. Wu, et al 2019 are shown in green. Dots outlined in red correspond to correlations with *p* < 0.05, after multiple hypothesis test corrections. (C) Correlation between mRNA stability and local secondary structure. For each ORF, the folding energy in 100 bp sliding windows was calculated, and the minimum value taken. Shown are boxplots for half-lives of endogenous HEK293T binned into quartiles by folding energy. Otherwise, as in A. (D) ORFeome mRNA stability does not correlate with local secondary structure. As in C, except for minimum folding energy. (E) microRNA-mediated regulation cannot explain the variation in ORFeome stability. ORFs were classified as containing or lacking seed-matched sites for the top five expressed mRNAs (site ORFs [orange] and no site ORFs [blue], respectively). Shown are boxplots for their half-lives for ORFeome cell lines 1 and 4 (top). Significance was calculated by the Kolmogorov-Smirnov test. (F) As in E, except for ORFeome decay rates from Q. Wu, et al. 2019. (G) AU-rich elements cannot explain the variation in ORFeome stability. As in E, except for AU-rich elements. (H) As in G, except for ORFeome decay rates from Q. Wu, et al. 2019. (I) AU-rich elements in ORFs destabilize mRNAs upon translational repression. As in G, except for half-lives determined in cell lines 1 and 4 in the presence of 4EGI-1.

**Figure S4. Amino acid use may affect mRNA stability and A-site dwell time.**

(A) Codons encoding the same amino acid can have similar relationships with mRNA stability. For each amino acid, shown are the average correlations of their average corresponding CSC score(s) (called amino acid stability coefficient or AASC), calculated from ORFeome datasets. Points represent the CSCs for the codon(s) encoding each amino acid. (B) Elongation speeds are influenced by the encoded amino acid. Shown are the average correlations, for each amino acid, of their elongation speed as determined in the HeLa rep 1 ribosome profiling library. Points represent the corresponding elongation speed for the codons encoding each amino acid. (C) HEK293T and HeLa cells have similar amino-acid-level elongation speeds. Plotted are elongation speeds for each amino acid in HeLa cells compared to HEK293T cells. (D) As in D, except comparing HeLa and U2OS amino acid elongation speeds. See also Tables S3, S4.
